# Nanoelectrode Atmospheric Pressure Chemical Ionization Mass Spectrometry

**DOI:** 10.1021/jasms.4c00117

**Published:** 2024-06-25

**Authors:** Nicole C. Auvil, Mark E. Bier

**Affiliations:** Department of Chemistry, Carnegie Mellon University, 4400 Fifth Avenue, Pittsburgh, Pennsylvania 15213, United States

**Keywords:** corona discharge, corona needle, atmospheric ionization, APCI, tungsten needle, gas-phase analysis, mass spectrometry

## Abstract

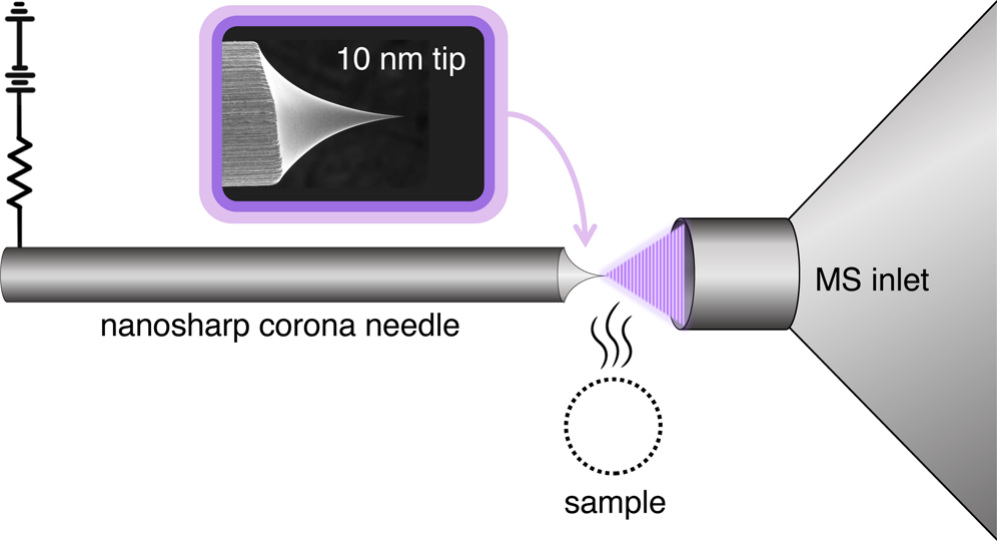

A small ionization needle with an ultrasharp, ultrafine tip is introduced. It is lab-fabricated from tungsten wire and serves as a corona discharge emitter in nanoelectrode atmospheric pressure chemical ionization mass spectrometry (nAPCI-MS). Tip radii ranged from 8 to 44 nm, up to 44× smaller than the sharpest previously reported corona needle. Because of this, nAPCI was able to operate at +1.0 kV with no auxiliary counter electrode. Alternatively, at +1.2 kV, nAPCI could be enclosed in a small plastic assembly for headspace analysis with a sampling tube attachment as long as 15 m. No added heat or gas flow was necessary. The efficacy of nAPCI-MS was demonstrated through needle durability studies and direct analysis of vapors from real-world samples. Provisional identifications include ibuprofen from a pharmaceutical tablet, albuterol aerosol sprayed from a medical inhaler, cocaine from paper currency, caffeine from a fingertip, and bisphenol E from a paper receipt.

## Introduction

Corona discharge radiates from the tip of a corona needle when sufficient voltage is applied. This energetic plasma ionizes gas-phase analytes, making it useful as the ion source of a mass spectrometer. The morphology of a corona needle affects its ionization efficiency. A needle with a smaller radius of curvature (ROC) is sharper and can produce corona discharge at a lower voltage.^1−4^ The ability to operate an ion source using lower voltage is desirable since it is safer for the instrument operator, uses less energy, has less risk of arcing to the instrument, and has greater practicality for implementation in portable instrumentation. A sharper corona needle also produces a physically smaller discharge which can allow for heightened spatial precision.^5^

Atmospheric pressure chemical ionization (APCI)^6^ and its variations including atmospheric solids analysis probe (ASAP),^7^ corona discharge atmospheric pressure ionization (CDAPI),^8^ and superatmospheric pressure chemical ionization (super-APCI)^9^ all use commercial APCI corona needles at +3–6 kV. Commercially available APCI needles typically do not have vendor-specified tip sharpness.

Efforts have been made in the past decade to use sharper needles for APCI mass spectrometry.^5,10−13^ As far as the authors are aware, the sharpest corona needle described for this purpose is a stainless steel acupuncture needle reported to have a tip diameter of 700 nm and therefore an ROC of 350 nm.^10−12^ The method of ROC measurement was not specified. In this study, we found acupuncture needles of the same brand and product number to have ROCs significantly larger than the reported value. These needles reportedly required +1.5–2.6 kV to operate under standard conditions and +3.4 kV when in a small plastic enclosure. A new needle was used at the beginning of each experiment or after 8 h of continuous use due to significant dulling.^10^

The next sharpest needle previously reported, a tungsten needle with an ROC of 500 nm, was used in a technique called nanotip ambient ionization mass spectrometry (NAIMS).^5^ The needle tip was positioned 20 μm above a copper plate counter electrode. The close proximity allowed for analysis of volatiles at only +1 kV. NAIMS needles dulled from 500 to 1350 nm ROC after 10 h of use (estimated using an optical microscope).

An ultrasharp, ultrafine, lab-fabricated corona needle is reported in this work. Needle tip morphology including ROC and cone height was quantified. The associated mass spectrometry ionization technique, nanoelectrode atmospheric pressure chemical ionization (nAPCI), was optimized. Needle durability was investigated. Direct analysis ability of nAPCI was demonstrated on vapors from solid, liquid, gaseous, and aerosolized samples.

## Experimental Section

### Needle Fabrication via Electrochemical Etching

**Figure 1:**
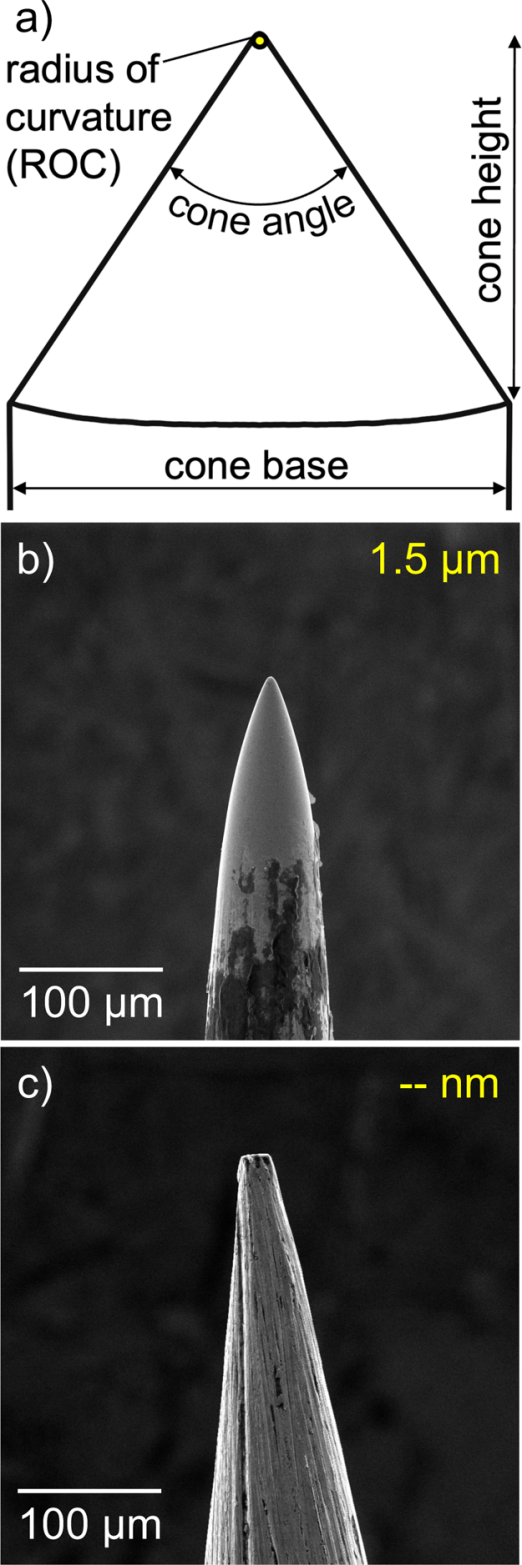
(a) Diagram of geometric parameters of a needle tip. (b) SEM image of an acupuncture corona needle tip. (c) SEM image of a commercial APCI corona needle tip. The ROC of each needle is given in yellow. ROC could not be determined for the commercial APCI needle because it did not have a curved tip.

Nanosharp tungsten corona needles were fabricated from 254 μm diameter tungsten wire using a modified version of the double electrolyte etching method reported by Li et al. in 2019.^14,15^ The circuit was designed to break at the precise moment needle formation is complete, preventing any further etching (and therefore dulling) of the sharp tip. A diagram of the electrochemical etching circuit can be found in Figure S1, and optical microscope images of the needle etching progression can be found in Figure S2. In past works, ROC and other needle tip morphology measurements, like those depicted in Figure 1a, have been estimated using optical microscope imaging. For a higher level of precision and accuracy when determining ROC in this study, scanning electron microscopy (SEM) imaging was used. ROCs were calculated from highest attainable magnification SEM image of each needle tip via the procedure in Figure S3.

### Ion Source

A freshly etched needle was positioned coaxially with the mass spectrometer’s heated ion transfer tube inlet at a distance less than or equal to 0.5 mm using a micromanipulator. A voltage of +1.0 kV was applied to the needle through a 400 MΩ resistor. Alternatively, the needle and inlet could be coupled and enclosed in a small plastic tee assembly, requiring a slightly higher voltage of +1.2 kV. The enclosure allowed for the attachment of a sampling tube with length up to 15 m. Vapors could be directly ionized by presenting a solid, liquid, gaseous, or aerosolized sample near the needle-inlet junction (in the exposed configuration) or at the sampling tube inlet (in the enclosed configuration). After formation in the corona discharge, ions entered the mass spectrometer inlet via pressure differential. No added gas flow, pumps, or heat sources were used.

### Instrumentation

SEM (Quanta 600 FEG, FEI Company, Eindhoven, Netherlands) was used for needle tip imaging. The settings were as follows: acceleration voltage: 10 kV, spot size: 2.0 or 3.0, dwell time: 3 μs, working distance: 10 mm, secondary electron detector.

nAPCI can be coupled with any atmospheric pressure ionization compatible mass spectrometer. Three mass spectrometers were used in this study, but an LTQ XL (Thermo Scientific, San Jose, CA) was the primary instrument used because of its large trapping volume and therefore high sensitivity.^16^ The settings were as follows: ion transfer tube temperature: 200 °C, ion transfer tube voltage: 50 V, tube lens: 100 V, microscans: 5, maximum ion inject time: 10 ms, scan range: 100–500 *m*/*z*, positive ion mode. An Exactive Plus EMR mass spectrometer (Thermo Scientific) was used for source geometry optimizations. The settings were as follows: ion transfer tube temperature: 220–260 °C, microscans: 10, maximum ion inject time: 50 ms, S lens radio frequency (RF) level: 200.0, scan range: 50–500 *m*/*z*, positive ion mode. An LCQ Deca XP Plus mass spectrometer (ThermoFisher Scientific, San Jose, CA) was used for a 5-day continuous outdoor air monitoring experiment. The settings were as follows: ion transfer tube temperature: 250 °C, ion transfer tube voltage: 13 V, tube lens offset: −30 V, microscans: 300, scan range: 15–200 and 50–2000 *m*/*z*, positive ion mode. For all mass spectrometers, optimal ion transfer tube temperature may vary based on thermal stability and volatility of analytes. A high voltage power supply was used to supply voltage to the needle (Bertan Associates Inc., Model 205B-10R, Valhalla, NY).

### Materials

Tungsten wire was purchased from World Precision Instruments (254 μm o.d., Sarasota, FL). The stainless steel washer used in needle etching measured 9.7 mm od, 4.0 mm id, 1.0 mm depth. NaCl and KOH used to make etching solutions were purchased from Fisher Scientific (Fair Lawn, NJ). Commercial APCI needles were obtained from Thermo Scientific (San Jose, CA). Stainless steel acupuncture needles were purchased from SEIRIN Company (type J, 120 μm o.d., Shizuoka, Japan). Ethylene tetrafluoroethylene (ETFE) tubing of 1.59 mm o.d. × 0.76 mm i.d. for needle sheath and 3.18 mm o.d. × 1.59 mm i.d. for sampling tube extension, a polyether ether ketone (PEEK) tee assembly of 1.59 mm i.d. with 1.25 mm through hole, and stainless steel tubing of 1.59 mm o.d. × 1.17 mm i.d. for the sampling tube were purchased from IDEX Health & Science (Oak Harbor, WA). Peppermint essential oil (Plant Therapy Inc. Twin Falls, ID) was purchased from a local grocery store. Ibuprofen tablets (200 mg, CVS Health, Woonsocket, RI) were purchased from a local CVS Pharmacy. An albuterol inhaler (ProAir HFA, 90 μg dose, IVAX Pharmaceuticals Ireland, Waterford, Ireland) was obtained from SpaceX. Receipt was obtained from Stephanie’s Café in the Mellon Institute building (Carnegie Mellon University, Pittsburgh, PA). ImageJ was used for calculating ROC from SEM images.

## Results and Discussion

### Needle Fabrication and Sharpness

**Figure 2:**
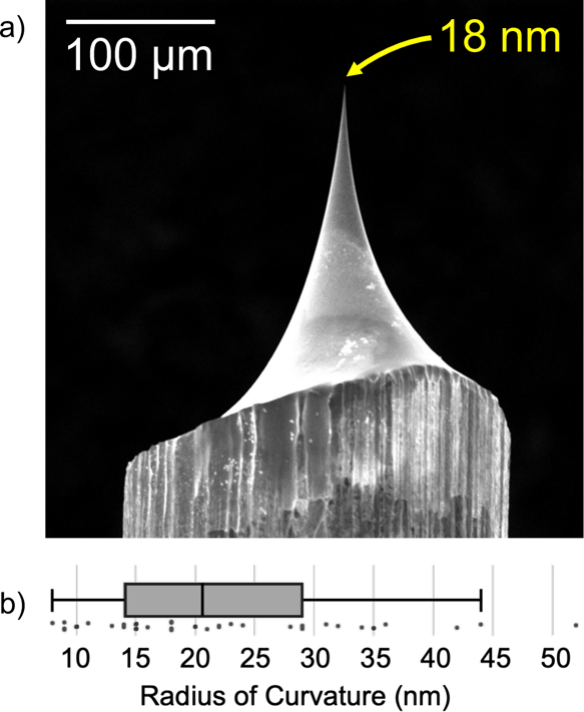
(a) SEM image of a nanosharp tungsten corona needle for nAPCI. ROC is given in yellow. (b) Box plot of nAPCI needle tip sharpness distribution, measured by ROC. Each data point is included beneath the box plot.

An electrochemically etched nAPCI needle tip is pictured in Figure 2a. SEM images of all 34 needles fabricated in this study can be found in Figure S4. The box plot in Figure 2b shows the distribution of needle sharpness. The sharpest had an 8 nm ROC, meaning that nAPCI needles are up to 44 times sharper than the sharpest previously reported corona needle.^10−12^ In different context, the width of a nAPCI needle tip can be as thin as ∼3 hemoglobin molecules or ∼64 tungsten atoms side by side.^17,18^ The dullest measured needle, 52 nm ROC, is a statistical outlier by Tukey’s method. Excluding it, the ROC range is 8 to 44 nm with a median of 21 nm. While this spread of sharpness is relatively narrow, it is expected that an etching setup with fewer positioning variables would reduce variability in needle sharpness. The needle’s short cone height is important for durability and safety, as the delicate portion is small and therefore less prone to accidental mechanical damage. Needles used previously in the lab had cone lengths of 1 cm or greater and were susceptible to damage.

The concave parabolic shape of nAPCI needle tips, graphically fit in Figure S5, is believed to allow for a level of sharpness that has not been achieved in convex ionization needle tips.^19,20^ The previous sharpest corona needle was a commercial stainless steel acupuncture needle reported to have an ROC of 350 nm.^10−12^ Pictured in Figure 1b, acupuncture needles of the same brand and product number were purchased and measured. They were found to have significantly larger ROCs at ∼1.5 μm; over 4× larger than the value reported in the literature. The reason for this discrepancy is unknown. A visual comparison can be found in Figure S6. Commercial APCI corona needles, pictured in Figure 1c, were found to have ROCs greater than or equal to 14 μm.

### Interface with the Instrument

nAPCI’s source geometry was optimized. Previous work determined that a corona needle positioned coaxially to and within 1 mm of a mass spectrometer inlet would maximize the needle tip apex electric field lines entering the mass spectrometer.^21^ Since the field lines produced by the tip apex have the highest electric field strength, this orientation maximizes ionization efficiency.^22^ This was confirmed to be true for nAPCI needles through voltage–response experiments. Coaxial needles were found to produce a stable corona discharge at a lower voltage than perpendicular needles. The distance between needle tip and mass spectrometer inlet was optimized to be 0.5 mm or less. This value may be smaller than determined in previous work because sharper needles produce physically smaller corona regions. These optimizations are shown graphically in Figure S7.

**Figure 3:**
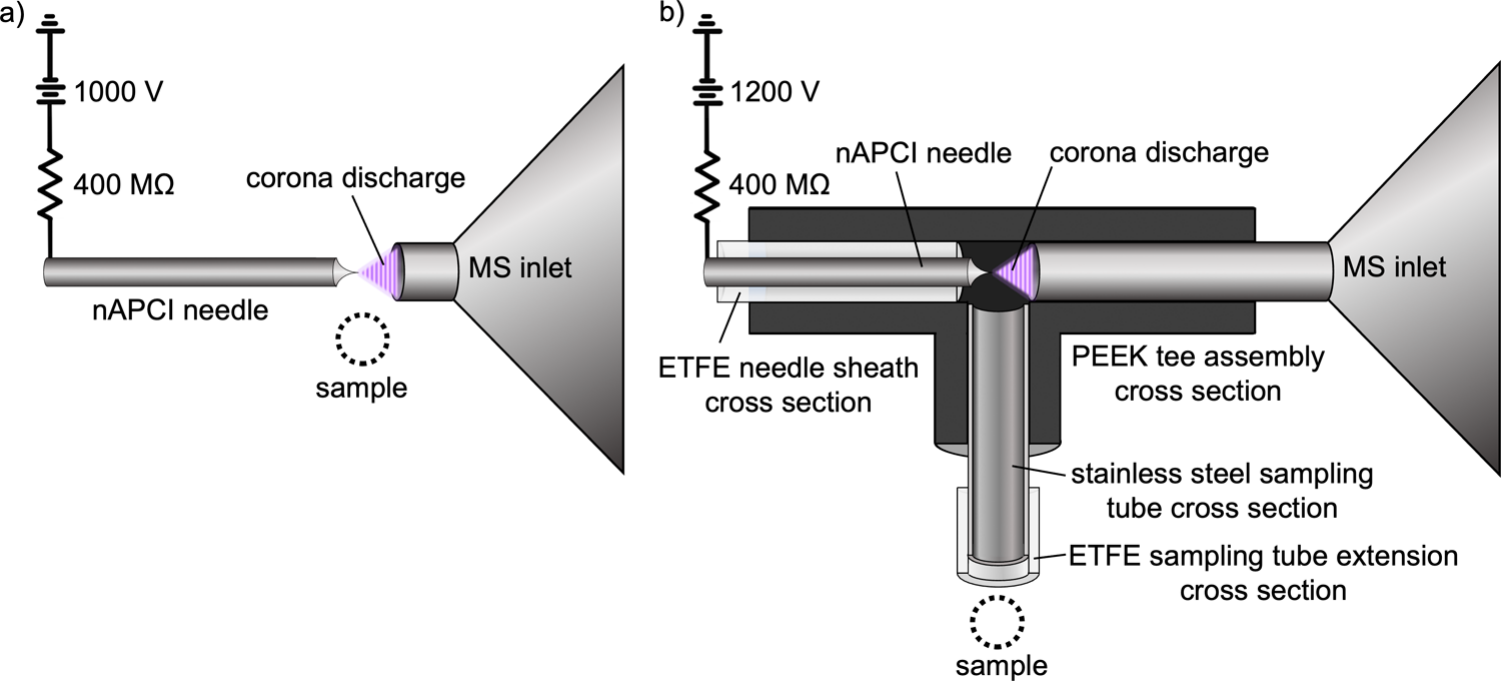
(a) Diagram of nAPCI in the exposed configuration. (b) Diagram of nAPCI in the enclosed configuration with sampling tube attachment up to 15 m. Not to scale.

nAPCI could be operated in an exposed configuration, with the needle held by a pin chuck, supported by a ring stand, and positioned with a micromanipulator, as depicted in Figure 3a. In this setup, the source could draw ambient air from all directions. If vapors from a solid or liquid sample were analyzed using the exposed configuration, the sample was held as close as possible to the discharge region, as optimized in Figure S7. The close proximity allowed for thermal energy from the heated ion transfer tube to aid analyte vaporization. Samples with greater analyte volatility may be presented at a greater distance.

Alternatively, nAPCI could be operated in an enclosed configuration in which the needle was housed within a PEEK tee assembly and held secure in a sheath of ETFE tubing, as depicted in Figure 3b. The opposite end of the tee was fit over a custom extended-length ion transfer tube. The distance between the needle tip and the ion transfer tube remained 0.5 mm or less within the assembly. This was accomplished using an optical microscope to view the needle-inlet junction through the open port in the tee during the needle insertion process. The enclosure blocked ambient air turbulence, only allowing the source to take in air from the open port. A sampling tube up to 15 m long could be attached to this port to allow for direct analysis of large, distant, or otherwise inaccessible samples. Vapors could also be directly analyzed from an enclosed headspace. The enclosed configuration’s assembly protects the needle tip, preventing accidental mechanical damage, and eliminates the need for a ring stand and micromanipulator to hold and position the needle.

### Electrifying the Needle

Previously reported corona discharge ion sources typically include a resistor between the power supply and the corona needle for the purpose of limiting arcing from the needle. In this work, voltage–response experiments were conducted using 1, 22, 200, and 400 MΩ resistors in the exposed configuration. The three lowest resistances resulted in electrical arcs, melting the needle tips. The 400 MΩ resistor successfully prevented arcing without limiting operating voltage or maximum signal intensity. For this reason, a 400 MΩ resistor was used for nAPCI. This resistance allows for a maximum current of 2.5 μA at 1000 V. Before and after SEM images of the needle tips can be found in Figure S8, along with total ion chromatograms of the voltage–response experiments.

**Figure 4:**
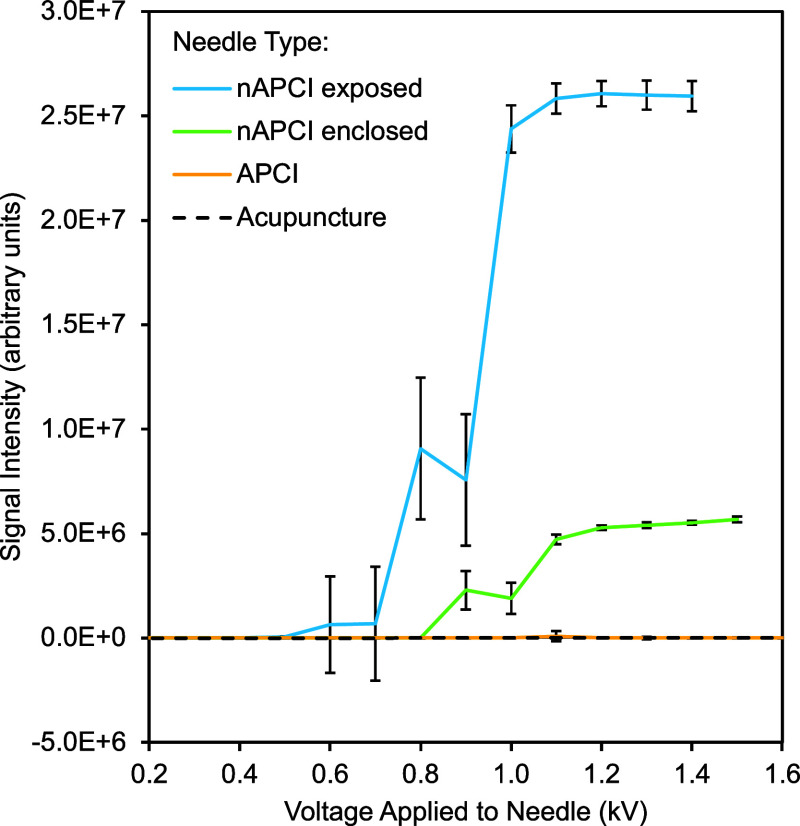
Voltage–response curves of an nAPCI needle in the exposed configuration, an nAPCI needle in the enclosed configuration, an acupuncture needle in the exposed configuration, and an APCI needle in the exposed configuration. Each voltage was held for 1 min before ramping up to the next 100 V. This period was averaged for each increment, with signal variability depicted via error bars (±1 standard deviation). TICs of these data can be found in Figure S9.

Operating voltage was determined experimentally via voltage–response experiments; the voltage at which the signal plateaus, maintaining a strong and stable intensity, indicates formation of a stable corona discharge. Figure 4 contains voltage–response curves. Each needle was exposed to up to +2.0 kV in increments of 100 V. The voltage at which signal intensity had a statistically significant plateau was determined to be the operating voltage. The commercial APCI needle and acupuncture needle were not able to generate stable corona discharge within this voltage range. The operating voltage of nAPCI in the exposed configuration was determined to be +1.0 kV. This remained true for needles anywhere in the full statistical range of nAPCI needle sharpness, as shown via in Figure S9. Therefore, variation in needle sharpness from the fabrication process was within tolerance of the source to produce a stable signal at +1.0 kV. The operating voltage of nAPCI in the enclosed configuration was determined to be +1.2 kV, 20% higher than that of the exposed configuration. This disparity is consistent with the findings of Habib and co-workers in 2013, whose corona needle enclosed in plastic tubing required 36% higher voltage than their open-air needle.^10^

**Figure 5:**
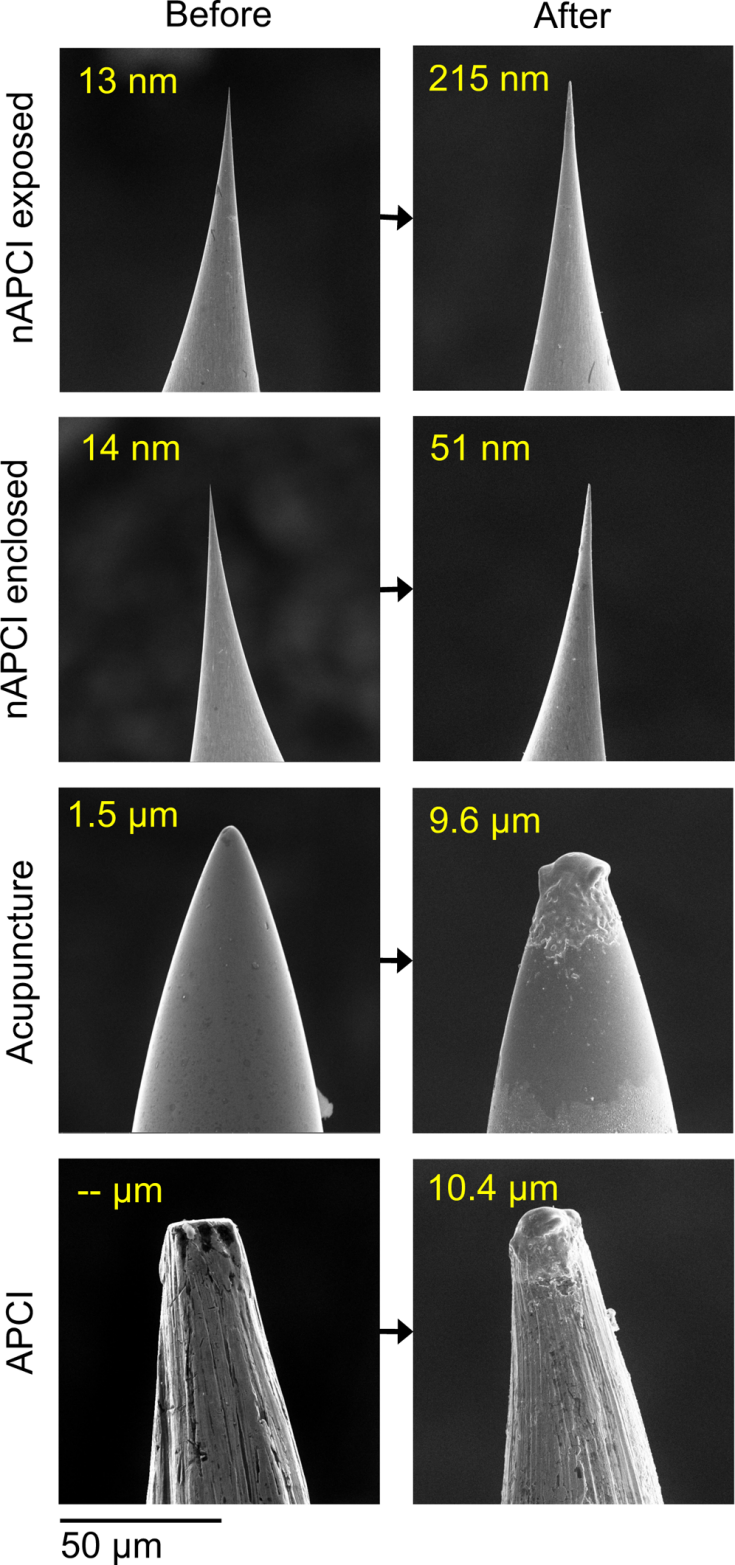
SEM images of corona needle tips before (left column) and after (right column) use in voltage–response experimentation. From top to bottom: nAPCI needle in the exposed configuration, nAPCI needle in the enclosed configuration, acupuncture needle in the exposed configuration, APCI needle in the exposed configuration. ROCs are given in yellow.

The corona needles used in voltage optimization experiments were imaged before and after voltage–response testing, as seen in Figure 5. The stainless steel acupuncture needle and APCI needle were significantly corroded and melted despite not having been exposed to high enough voltage to produce a stable corona discharge. Their resulting ROCs were both ∼10 μm. The tungsten nAPCI needles, in both configurations, exhibited far less dulling. They appeared to be relatively resistant to corrosion and melting despite their fine tips. However, their ROC change was significant enough to require a higher voltage to restart corona discharge after being turned off (shown in Figure S9f–h), so a new nAPCI needle was used at the beginning of each experiment session.

### Ion Source Performance

**Figure 6:**
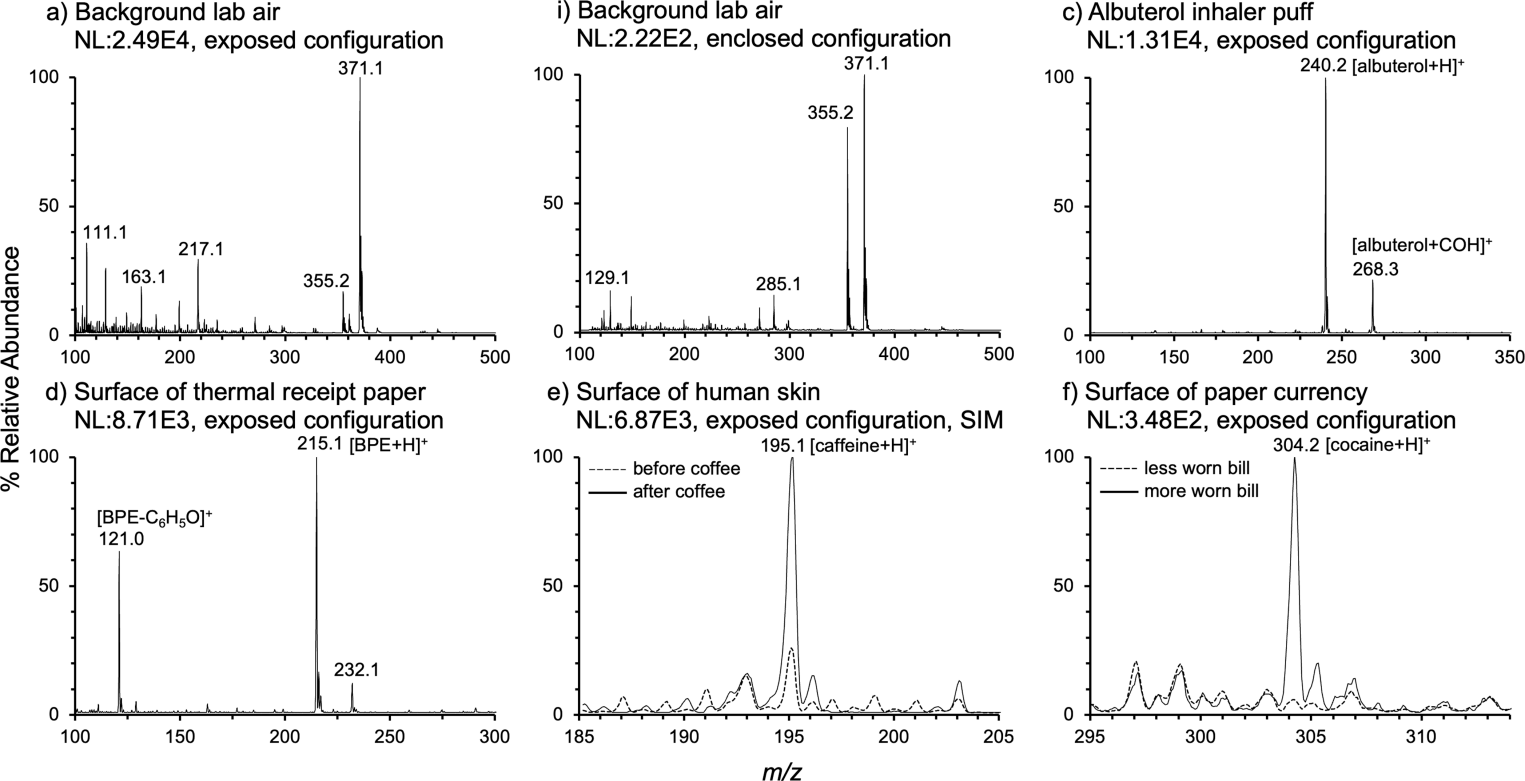
Mass spectra of various samples analyzed by nAPCI-MS. (a) Background lab air collected using the exposed configuration. (b) Background lab air collected using the enclosed configuration. The remaining four spectra were all collected using the exposed configuration. (c) Albuterol from a single inhaler puff. (d) Bisphenol E from the surface of thermal receipt paper. (e) Caffeine from the surface of human skin. (f) Cocaine from the surface of paper currency. All spectra were collected in normal scan mode with a mass range of 100–500 *m*/*z*, except for spectrum e, which was collected in selected ion monitoring (SIM) mode. Each mass spectrum was averaged from between 5 and 60 spectra. All spectra were background subtracted and offset from the *x*-axis by 1% for clarity.

**Figure 7:**
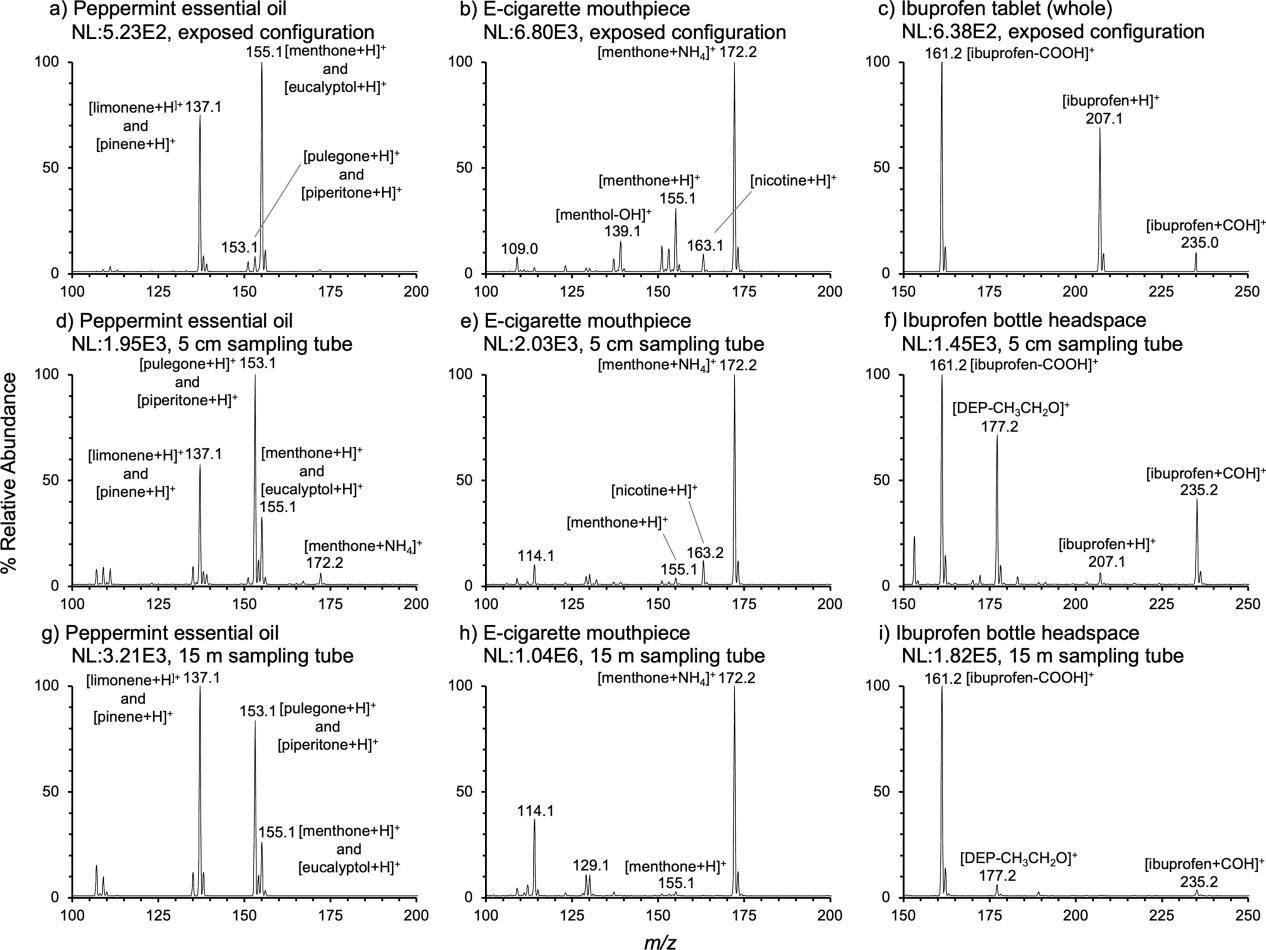
Mass spectra of three samples analyzed by nAPCI-MS with varying sampling tube length. (a–c) Collected using the exposed configuration (no sniffing tube). (d–f) Collected using the enclosed configuration with a 5 cm stainless steel sampling tube. (g–i) Collected using the enclosed configuration with a 15 m ETFE sampling tube extension. The analyzed samples were peppermint essential oil, an e-cigarette mouthpiece, and an ibuprofen tablet/headspace of an ibuprofen bottle in the left, middle, and right columns, respectively. All spectra were collected in normal scan mode with a mass range of 100–500 *m*/*z*. Each mass spectrum was averaged from between 5 and 60 spectra. All spectra were background subtracted and offset from the *x*-axis by 1% for clarity.

The direct ionization capability of nAPCI was demonstrated on analytes ranging in volatility. Figures 6 and 7 contain the resulting spectra with provisional peak assignments. Figures 6a and 6b are spectra of background lab air collected in the exposed configuration and the enclosed configuration, respectively. Figure 6c is a spectrum of albuterol from a single inhaler puff sprayed directly at the exposed nAPCI source from ∼8 cm away. Each puff is ∼1 s in length. The most abundant ion detected was [albuterol + H]^+^ at *m*/*z* 240.2.^24^

Thermal receipt paper has historically been coated with the endocrine disrupting chemical bisphenol A (BPA). Health concerns have resulted in regulation of BPA for this use, but manufacturers have turned to structurally similar chemicals with potentially similar health impacts.^30,31^ The replacement coating bisphenol E (BPE) was detected by holding the surface of a shopping receipt up to the exposed nAPCI source and collecting the mass spectrum in Figure 6d. The most abundant ion detected was [BPE + H]^+^ at *m*/*z* 215.1.^25^ A presumed fragment was also identified at *m*/*z* 121.0.^26^

Caffeine was detected from the surface of human skin in Figure 6e. An ungloved finger was held near the exposed nAPCI source for analysis using selected ion monitoring (SIM) mode with a scan window of 20 Da bracketing the protonated caffeine ion at *m*/*z* 195.1. The subject had not consumed caffeine in the 24 h prior to experiment start. The initial finger scan showed low level detection of [caffeine + H]^+^ or an isobaric ion. The subject then drank a cup of coffee. Thirty minutes later, enough time for caffeine to enter the bloodstream, the subject’s finger was scanned again and the [caffeine + H]^+^ peak was ∼4× more abundant.^27^ The detected caffeine was presumed to have exited the subject’s bloodstream through their pores, as caffeine is known to effectively pass through human skin.^28^

Similarly, cocaine was detected from the surface of paper currency. It is known that a large percentage of American paper currency carries traces of cocaine, and direct analysis in real time (DART) mass spectrometry has previously been used to screen bills for the drug.^29,30^ The edge of a moderately worn bill that had been in circulation was scanned ∼1 mm away from the nAPCI source, and [cocaine + H]^+^ at *m*/*z* 304.2 was observed, as seen in Figure 6f. Overlaid extracted ion chromatograms of cocaine and its characteristic fragment at *m*/*z* 182 are given in Figure S10. The presence of these two ions is aligned temporally, providing further confidence in the identification of cocaine. The authors attempted to obtain an uncirculated bill to use as a control, but one could not be obtained at the time of experimentation. Instead, a bill that had been in circulation but did not look heavily used was tested. Cocaine was not detected on this bill above the noise level.

The spectrum in Figure 7a shows the aromatic profile of peppermint essential oil. A vial was opened ∼15 cm beneath the exposed nAPCI source, producing a mass spectrum in which the most abundant ion was [menthone + H]^+^ and/or [eucalyptol + H]^+^ at *m*/*z* 155.1, followed by [limonene + H]^+^ and/or [pinene + H]^+^ at *m*/*z* 137.1. At a lower relative abundance, [pulegone + H]^+^ and/or [piperitone + H]^+^ at *m*/*z* 153.1 were/was detected. These sets of structural isomers are volatile organic terpenes known for their fresh scent.^31,32^

nAPCI-MS was used to analyze another minty scent profile, the volatiles from a “cool mint” flavored electronic cigarette (e-cigarette). The e-cigarette mouthpiece was held ∼1 cm from the exposed ion source. Aerosol production was not induced; only the molecules passively diffusing from the device were analyzed. As seen in Figure 7b, the most abundant ion in the spectrum was [C_10_H_22_ON]^+^, followed by [menthone + H]^+^ and [menthol – OH]^+^.^32^ The less volatile [nicotine + H]^+^ at *m*/*z* 163.2 was also detected.^33^ This is consistent with the product packaging, which lists nicotine with menthol as flavoring. Menthol oxidizes into the more volatile menthone.^34^ The identity of the [C_10_H_22_ON]^+^ ion may be [menthone + NH_4_]^+^ due to its apparent high volatility. It has been labeled as such in Figure 7. Ammonia has been utilized by the tobacco industry in the synthesis of freebase nicotine for inhalation, a process which produces ammonium as a side product.^35,36^

Figure 7c was produced by an ibuprofen tablet held ∼1 mm from the exposed source for a few seconds. The [ibuprofen + H]^+^ ion was present at *m*/*z* 207.1, and its characteristic fragment [ibuprofen – COOH]^+^ also had a prominent abundance.^23^ All samples were undamaged by nAPCI and could theoretically be consumed/used after analysis. Baseline signal remained consistent throughout the 45 min experimental session with nAPCI in the exposed configuration (TIC in Figure S11).

The enclosed configuration was tested with three different samples at two different sampling tube lengths: a 5 cm stainless steel sampling tube (Figure 7d–f) and a 15 m ETFE sampling tube extension slip fit over the shorter tube (Figure 7g–i). Both sampling tubes were unheated and had no added pumps. An open vial of peppermint essential oil was presented at the sampling tube inlet to produce Figures 7d and 7g. They contain the same major terpene peaks found using the exposed configuration (no sampling tube). However, as the sampling tube length was increased from 5 cm to 15 m, the relative abundance of the lower molecular weight terpenes increased while the relative abundance of the higher molecular weight terpenes decreased. This may be attributed to their relative volatilities and adsorption to the inner surface of the tubing.

The e-cigarette previously analyzed by nAPCI-MS’s exposed configuration was analyzed using the enclosed configuration in Figures 7e and 7h. The e-cigarette was not turned on and thus was not producing aerosol; its mouthpiece was simply held near the sampling tube inlet. Once again, the same major peaks were present, but their relative abundances varied with sampling tube length. Most notably, [menthone + NH_4_]^+^ increased in relative abundance, while [menthone + H]^+^ and [menthol – OH]^+^ decreased in relative abundance as sampling tube length increased. The less volatile [nicotine + H]^+^ decreased in relative abundance with increasing tube length, but absolute abundance went down by 60% from null to 5 cm and up by 944% from 5 cm to 15 m.

Finally, the open headspace of a 500-tablet ibuprofen bottle was analyzed in the enclosed configuration. The inlet of the sampling tube was inserted ∼4 cm into the mouth of the open bottle and the bottle was gently shaken side to side. The plasticizer diethyl phthalate (DEP) was identified in the form of its characteristic fragment, [DEP – CH_3_CH_2_O]^+^ at *m*/*z* 177.2.^37^ Phthalate plasticizers are known to be used in pharmaceutical pill coatings and packaging.^38^Figures 7f and 7i show that the relative abundance of [ibuprofen + H]^+^ and [ibuprofen + COH]^+^ decreased, while [ibuprofen – COOH]^+^ increased with sampling tube length. Following the same trend as nicotine, the absolute abundance of [ibuprofen + H]^+^ went down by 81% from null to 5 cm and up by 582% from 5 cm to 15 m. Significant signal increase and ion intensity ratio changes were observed when using the 15 m tube for all samples. This may be attributed to a lower pressure in the corona discharge region resulting in a brighter ion source due to Paschen’s Law and/or resulting improved efficiency of ion intake through the ion transfer tube.

Continuous usage capability of nAPCI was examined through extended air monitoring experiments. Both the exposed and enclosed configurations were left to continuously monitor ambient laboratory air for 20 h. Figure S12 contains the resulting TICs. The enclosed configuration produced a less noisy signal, presumably due to its enclosure blocking air turbulence. Ambient air, even indoors, is constantly changing chemical composition due to factors such ventilation turbulence, humidity, and dynamic volatile emissions. Because ambient laboratory air is the background signal for nAPCI analysis, both configurations have baselines that may change over the course of experimental use. While this may limit nAPCI’s use for quantitative analysis in its current form, the use of ambient air as a reagent gas is cost free and versatile.

The 20-h signal monitoring experiments were started at offset times of a day, yet analogous changes in signal occurred at the same time of day for both experiments. This suggests the cause is changes in the lab air (e.g., air conditioning turning on/off, custodian passing through on schedule), rather than changes in ionization capabilities of the needle caused by usage time. To further test durability, nAPCI was used to monitor outdoor air organic chemicals for 5 days continuously during the Canadian wildfires of June 2023. There was no significant drop in performance (TIC in Figure S13).

## Conclusion

Nanoelectrode-APCI was introduced and optimized. The short, electrochemically etched corona needle tips had radii as small as 8 nm, making them 44× sharper than the sharpest previously reported corona needle. This allowed the needles to produce stable corona discharge at a DC voltage of +1.0 kV with no auxiliary counter electrode. nAPCI could be used in the open air or enclosed in a small assembly with an unheated sampling tube up to 15 m long. The source’s direct analysis capabilities were demonstrated on small vapor molecules from real world samples including cocaine from paper currency, bisphenol E coating from receipt paper, caffeine from human skin, albuterol aerosol from an inhaler, nicotine from an e-cigarette, ibuprofen from unbroken tablets, and terpenes from peppermint essential oil. Given the low volatility of some of these analytes, nAPCI appears to have a relatively high ionization efficiency despite its small discharge region. The samples were not damaged by analysis. The 20-h and 5-day air monitoring experiments illustrated nAPCI’s robust continuous usage capabilities. The small, lightweight, low voltage nature of this simple ion source makes it suitable for portable instrumentation. Additionally, nAPCI may be ideal for high resolution ambient imaging mass spectrometry due to the spatial precision of its physically small discharge.

## Supporting Information Available

Diagram of nAPCI needle etching circuit (Figure S1). Optical microscope images of needle etching progression (Figure S2). Method for determining needle tip ROC (Figure S3). SEM images of all nAPCI corona needles fabricated for this study (Figure S4). Graphical parabolic fit of needle tips (Figure S5). SEM images showing the sharpness of SEIRIN Type J acupuncture needles (Figure S6). nAPCI-MS geometry optimizations (Figure S7). Voltage titrations for determining what resistance to use in nAPCI circuit (Figure S8). Voltage–response experiment TICs (Figure S9). Overlaid EICs of cocaine and characteristic fragment of cocaine (Figure S10). TIC of a 45 min experimental session using nAPCI exposed configuration (Figure S11). TICs of a 20-h indoor air monitoring using nAPCI in both configurations (Figure S12). TIC of 5-day outdoor air monitoring using nAPCI enclosed configuration (Figure S13) (PDF)

## References

[ref1] Wen T. Y.; Su J. L. Corona Discharge Characteristics of Cylindrical Electrodes in a Two-Stage Electrostatic Precipitator. Heliyon 2020, 6 (2), e03334 10.1016/j.heliyon.2020.e03334. 32095646 PMC7033520

[ref2] Boltachev G. S.; Zubarev N. M. Analytical Model of a Corona Discharge from a Conical Electrode under Saturation. Technical Physics 2012 57:11 2012, 57 (11), 1493–1502. 10.1134/S1063784212110084.

[ref3] Intra P.; Yawootti A.; Rattanadecho P. Influence of the Corona-Wire Diameter and Length on Corona Discharge Characteristics of a Cylindrical Tri-Axial Charger. J. Electrostat 2015, 74, 37–46. 10.1016/j.elstat.2014.12.002.

[ref4] Intra P.; Tippayawong N. Effect of Needle Cone Angle and Air Flow Rate on Electrostatic Discharge Characteristics of a Corona-Needle Ionizer. J. Electrostat 2010, 68 (3), 254–260. 10.1016/j.elstat.2010.01.008.

[ref5] Zhou Z.; Lee J. K.; Kim S. C.; Zare R. N. Nanotip Ambient Ionization Mass Spectrometry. Anal. Chem. 2016, 88, 5542–5548. 10.1021/acs.analchem.6b01212. 27087600

[ref6] Carroll D. I.; Dzidic I.; Stillwell R. N.; Haegele K. D.; Horning E. C. Atmospheric Pressure Ionization Mass Spectrometry. Corona Discharge Ion Source for Use in a Liquid Chromatograph-Mass Spectrometer-Computer Analytical System. Anal. Chem. 1975, 47 (14), 2369–2373. 10.1021/ac60364a031.

[ref7] McEwen C. N.; McKay R. G.; Larsen B. S. Analysis of Solids, Liquids, and Biological Tissues Using Solids Probe Introduction at Atmospheric Pressure on Commercial LC/MS Instruments. Anal. Chem. 2005, 77 (23), 7826–7831. 10.1021/ac051470k. 16316194

[ref8] Liang H. Z.; Zhang X.; Chen S. X.; Shao Z. Corona Discharge Atmospheric Pressure Ionization Mass Spectrometry for Real Time Gas Analysis. Chinese Journal of Analytical Chemistry 2008, 36 (8), 1152–1156. 10.1016/S1872-2040(08)60062-6.

[ref9] Chen L. C.; Rahman M. M.; Hiraoka K. Super-Atmospheric Pressure Chemical Ionization Mass Spectrometry. Journal of Mass Spectrometry 2013, 48 (3), 392–398. 10.1002/jms.3173. 23494797

[ref10] Habib A.; Usmanov D.; Ninomiya S.; Chen L. C.; Hiraoka K. Alternating Current Corona Discharge/Atmospheric Pressure Chemical Ionization for Mass Spectrometry. Rapid Commun. Mass Spectrom. 2013, 27 (24), 2760–2766. 10.1002/rcm.6744. 24214861

[ref11] Rahman M. M.; Jiang T.; Tang Y.; Xu W. A Simple Desorption Atmospheric Pressure Chemical Ionization Method for Enhanced Non-Volatile Sample Analysis. Anal. Chim. Acta 2018, 1002, 62–69. 10.1016/j.aca.2017.11.033. 29306414

[ref12] Usmanov D. T.; Hiraoka K.; Wada H.; Matsumura M.; Sanada-Morimura S.; Nonami H.; Yamabe S. Non-Proximate Mass Spectrometry Using a Heated 1-m Long PTFE Tube and an Air-Tight APCI Ion Source. Anal. Chim. Acta 2017, 973, 59–67. 10.1016/j.aca.2017.03.044. 28502428

[ref13] Song L.; You Y.; Evans-Nguyen T. Surface Acoustic Wave Nebulization with Atmospheric-Pressure Chemical Ionization for Enhanced Ion Signal. Anal. Chem. 2019, 91 (1), 912–918. 10.1021/acs.analchem.8b03927. 30481449

[ref14] Li Y.; Song Z.; Zhang Y.; Wang Z.; Xu Z.; Lin R.; Qian J. A Double-Electrolyte Etching Method of High-Quality Tungsten Probe for Undergraduate Scanning Tunneling Microscopy and Atomic Force Microscopy Experiments. Eur. J. Phys. 2019, 40 (2), 025004 10.1088/1361-6404/aaf5e4.

[ref15] Li B.; Zhang Y.; Wang J.; Jia Z.; Shi C.; Ma Y.; Ma L. Fabricating Ultra-Sharp Tungsten STM Tips with High Yield: Double-Electrolyte Etching Method and Machine Learning. SN Appl. Sci. 2020, 10.1007/s42452-020-3017-4.

[ref16] Bier M. E.; Syka J. E. P. Ion Trap Mass Spectrometer System and Method. 5420425, May 30, 1995.

[ref17] Erickson H. P. Size and Shape of Protein Molecules at the Nanometer Level Determined by Sedimentation, Gel Filtration, and Electron Microscopy. Biol. Proced Online 2009, 11 (1), 32 10.1007/s12575-009-9008-x. 19495910 PMC3055910

[ref18] Li X.; Xie Y.; Nie Y.; Peng H. Influence of Pressure on the Valence Bond Structure of Tungsten. Physica B Condens Matter 2007, 394 (1), 27–32. 10.1016/j.physb.2007.02.017.

[ref19] Yamaguchi T.; Inami E.; Goto Y.; Sakai Y.; Sasaki S.; Ohno T.; Yamada T. K. Fabrication of Tungsten Tip Probes within 3 s by Using Flame Etching. Rev. Sci. Instrum. 2019, 90 ( (6), ). 10.1063/1.5085251. 31254980

[ref20] Chang W. T.; Hwang I. S.; Chang M. T.; Lin C. Y.; Hsu W. H.; Hou J. L. Method of Electrochemical Etching of Tungsten Tips with Controllable Profiles. Rev. Sci. Instrum. 2012, 83 ( (8), ). 10.1063/1.4745394. 22938300

[ref21] Sabo M.; Matejčík Š. A Corona Discharge Atmospheric Pressure Chemical Ionization Source with Selective NO + Formation and Its Application for Monoaromatic VOC Detection. Analyst 2013, 138 (22), 6907–6912. 10.1039/c3an00964e. 24081306

[ref22] Sekimoto K.; Takayama M. Negative Ion Formation and Evolution in Atmospheric Pressure Corona Discharges between Point-to-Plane Electrodes with Arbitrary Needle Angle. European Physical Journal D 2010, 60 (3), 589–599. 10.1140/epjd/e2010-10449-7.

[ref23] Zayed M. A.; Hawash M. F.; Fahmey M. A.; El-Gizouli A. M. M. Investigation of Ibuprofen Drug Using Mass Spectrometry, Thermal Analyses, and Semi-Empirical Molecular Orbital Calculation. J. Therm Anal Calorim 2012, 108 (1), 315–322. 10.1007/s10973-011-1876-z.

[ref24] Ye F.; Liu S.; Yang Y.; Zhao T.; Li S.; Zhou T.; Tan W. Identification of the Major Metabolites of (R)-Salbutamol in Human Urine, Plasma and Feces Using Ultra High Performance Liquid Chromatography Coupled with Quadrupole Time-of-Flight Mass Spectrometry. J. Sep Sci. 2019, 42 (20), 3200–3208. 10.1002/jssc.201900330. 31389651

[ref25] Frankowski R.; Zgoła-Grześkowiak A.; Grześkowiak T.; Sójka K. The Presence of Bisphenol A in the Thermal Paper in the Face of Changing European Regulations - A Comparative Global Research. Environ. Pollut. 2020, 265, 114879 10.1016/j.envpol.2020.114879. 32505936

[ref26] Moid AlAmmari A.; Rizwan Khan M.; Aqel A. Trace Identification of Endocrine-Disrupting Bisphenol A in Drinking Water by Solid-Phase Extraction and Ultra-Performance Liquid Chromatography-Tandem Mass Spectrometry. J. King Saud Univ Sci. 2020, 32 (2), 1634–1640. 10.1016/j.jksus.2019.12.022.

[ref27] Mort J. R.; Kruse H. R. Timing of Blood Pressure Measurement Related to Caffeine Consumption. Ann. Pharmacother 2008, 42 (1), 105–110. 10.1345/aph.1K337. 18094346

[ref28] Trauer S.; Patzelt A.; Otberg N.; Knorr F.; Rozycki C.; Balizs G.; Büttemeyer R.; Linscheid M.; Liebsch M.; Lademann J. Permeation of Topically Applied Caffeine through Human Skin - a Comparison of in Vivo and in Vitro Data. Br. J. Clin. Pharmacol. 2009, 68 (2), 181 10.1111/j.1365-2125.2009.03463.x. 19694736 PMC2767280

[ref29] Zuo Y.; Zhang K.; Wu J.; Rego C.; Fritz J. An Accurate and Nondestructive GC Method for Determination of Cocaine on US Paper Currency. J. Sep Sci. 2008, 31 (13), 2444–2450. 10.1002/jssc.200800117. 18646272

[ref30] JEOL USA. Instantaneous Detection of Illicit Drugs on Currency; JEOL, Peabody, MA, 2020. www.jeol.com.

[ref31] Rohloff J. Monoterpene Composition of Essential Oil from Peppermint (Mentha x Piperita L.) with Regard to Leaf Position Using Solid-Phase Microextraction and Gas Chromatography/Mass Spectrometry Analysis. J. Agric. Food Chem. 1999, 47 (9), 3782–3786. 10.1021/jf981310s. 10552722

[ref32] Gisler A.; Lan J.; Singh K. D.; Usemann J.; Frey U.; Zenobi R.; Sinues P. Real-Time Breath Analysis of Exhaled Compounds upon Peppermint Oil Ingestion by Secondary Electrospray Ionization-High Resolution Mass Spectrometry: Technical Aspects. J. Breath Res. 2020, 14 (4), 046001 10.1088/1752-7163/ab9f8b. 32691749

[ref33] Forbes T. P.; Krauss S. T. Confined DART-MS for Rapid Chemical Analysis of Electronic Cigarette Aerosols and Spiked Drugs. J. Am. Soc. Mass Spectrom. 2021, 32 (8), 2274–2280. 10.1021/jasms.1c00103. 34184882 PMC9969341

[ref34] Schmitz D.; Shubert V. A.; Betz T.; Schnell M. Exploring the Conformational Landscape of Menthol, Menthone, and Isomenthone: A Microwave Study. Front Chem. 2015, 3 (Mar), 132365 10.3389/fchem.2015.00015. PMC4355985 25815287

[ref35] Pankow J. F.; Mader B. T.; Isabelle L. M.; Luo W.; Pavlick A.; Liang C. Conversion of Nicotine in Tobacco Smoke to Its Volatile and Available Free-Base Form through the Action of Gaseous Ammonia. Environ. Sci. Technol. 1997, 31 (8), 2428–2433. 10.1021/es970402f.

[ref36] Kimbrough D. Vaping: What You Need to Know. American Chemical Society ChemMatters; ACS, 2019; p 5–8.

[ref37] Wang W.; Kannan K. Leaching of Phthalates from Medical Supplies and Their Implications for Exposure. Environ. Sci. Technol. 2023, 57 (20), 7675–7683. 10.1021/acs.est.2c09182. 37154399 PMC10210534

[ref38] Sree C. G.; Buddolla V.; Lakshmi B. A.; Kim Y. J. Phthalate Toxicity Mechanisms: An Update. Comparative Biochemistry and Physiology Part C: Toxicology & Pharmacology 2023, 263, 109498 10.1016/j.cbpc.2022.109498. 36374650

