# Supplementary material for: Nanoelectrode Atmospheric Pressure Chemical Ionization Mass Spectrometry
Source: J Am Soc Mass Spectrom. 2024 Jun 25;35(10):2288–96. doi: 10.1021/jasms.4c00117 (PMC11450814; doi:10.1021/jasms.4c00117)
Supplement: Supplementary file 1 — js4c00117_si_001.pdf [file js4c00117_si_001.pdf]

Supporting Information

Nanoelectrode Atmospheric Pressure Chemical Ionization Mass Spectrometry

Nicole C. Auvil[a] and Mark E. Bier*[a]
[a]Department of Chemistry, Carnegie Mellon University, 4400 Fifth Avenue, Pittsburgh, Pennsylvania 15213, United States
*Correspondence: mbier@cmu.edu

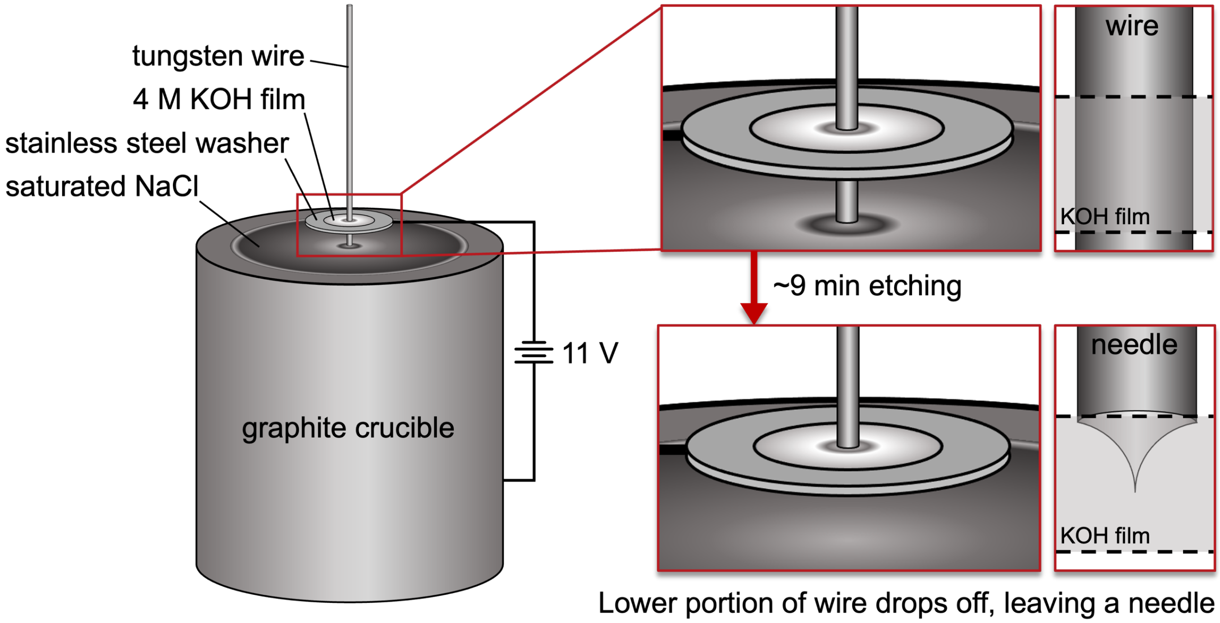

Figure S1. Diagram of nAPCI needle etching circuit. A graphite crucible filled with saturated NaCl solution was connected to the positive pole of a DC power supply. A 4 M KOH film in a stainless-steel washer connected to the power supply's negative pole was suspended above the NaCl solution. A length of 254 µm diameter solid tungsten wire was inserted through the film until it touched the NaCl solution, completing the circuit. The wire was centered in the washer by eye. The length of wire protruding beneath the film was kept less than ~2 mm to minimize wire mass below the film. A ring stand suspended the tungsten wire and the washer. Electrochemical etching occurred in the KOH film, where the tungsten wire was the anode, and the stainless-steel washer was the cathode. As the solid tungsten became oxidized and departed the wire's surface as tungstate, the wire became increasingly thin. The wire's lower portion dropped off when its weight overcame the tensile strength of the thinly etched portion. This took about 9 minutes. After etching, needles were rinsed with DI water to remove KOH crystals. The setup remained at uncontrolled room temperature. Inset shows a zoom-in and cross section of the etching region before etching begins and after etching is completed. Not to scale.

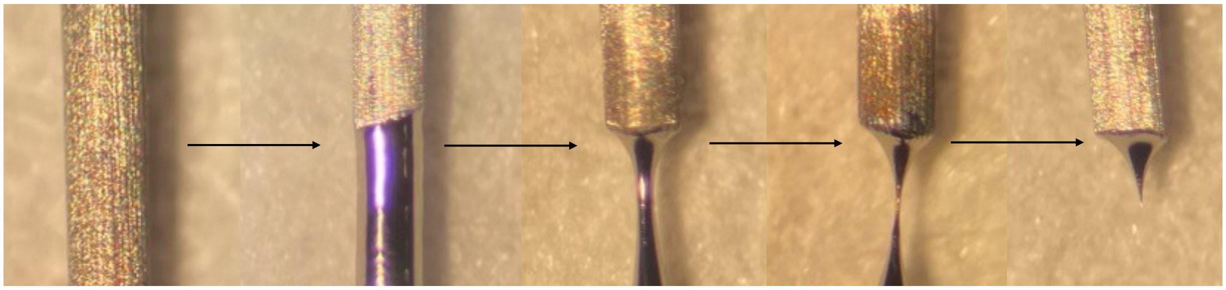

Figure S2. Optical microscope images of needle etching progression. 60X magnification. Starting material: 254 μm tungsten wire.

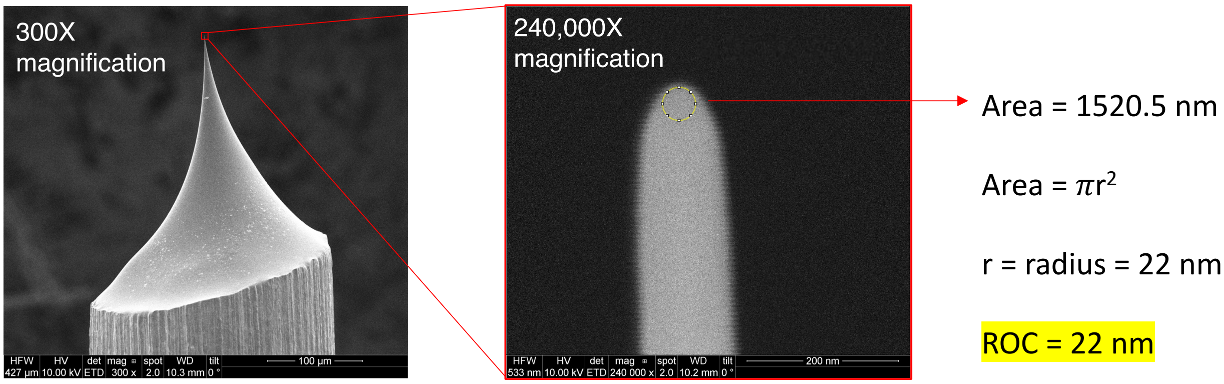

Figure S3. Needle tip was imaged using SEM at three magnifications: 300X, 1000X, and 240kX or the highest magnification at which the entire tip was visible. The two lower magnifications produced images useful for examining full needle tip morphology, while the highest magnification produced images of the needle tip's ultra-sharp point. The latter was used to calculate ROC. Method for determining needle tip radius of curvature (ROC):

1. Open SEM image in ImageJ (free image processing program).
   a) The image should be at the highest magnification possible while remaining in focus and containing the entire curvature of the tip.
2. Using line tool, draw a line the length of the scale bar.
3. Analyze > Set Scale. Input known distance and unit of length. Hit Ok.
4. Using oval selection tool, draw a circle at the very tip, following the curvature.
5. Click M key on keyboard to measure the area of the circle.
6. Find radius from area using $A = \pi r^2$
7. r = ROC

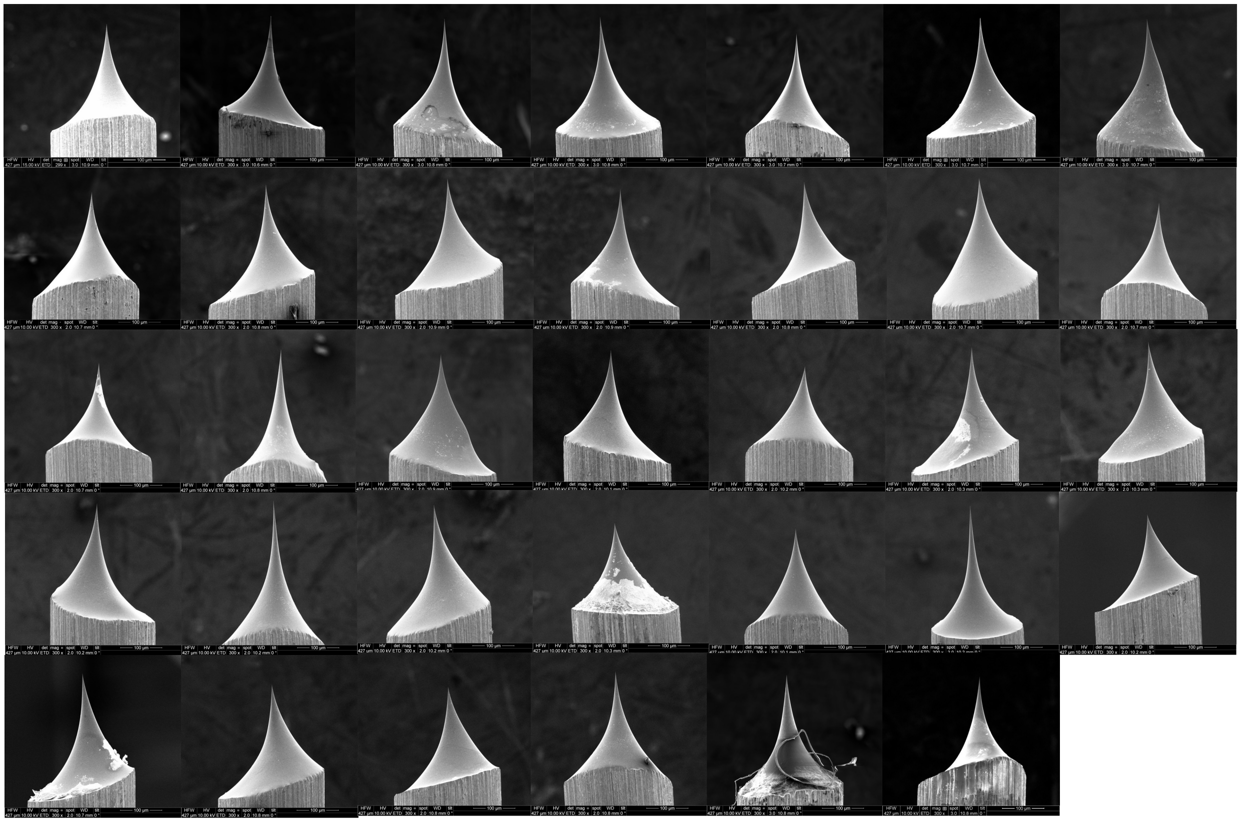

Figure S4. SEM images of all nAPCI needles fabricated via electrochemical etching in this work. 300X magnification.

	Parabolic ($y=ax^2$)	Hyperbolic ($[(y^2/a^2)-(x^2/b^2)]=1$)
tip apex curvature	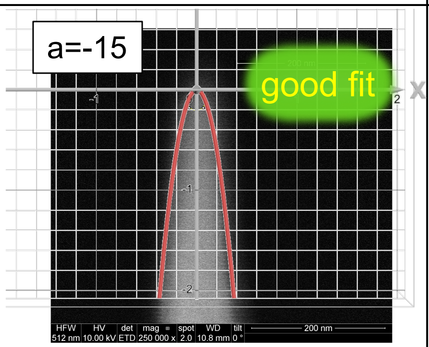	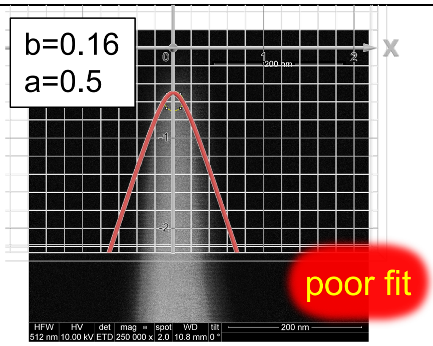
tip profile negative space curvature	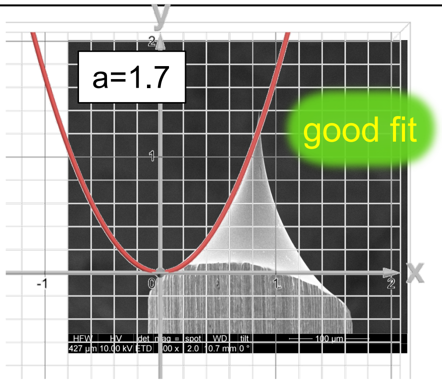	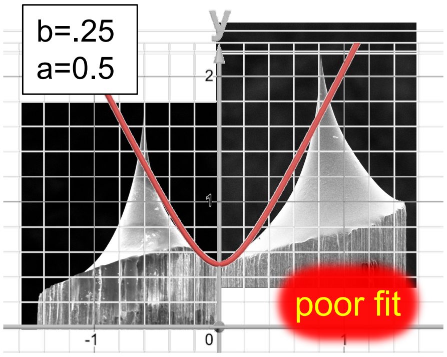

Figure S5. There are two ways in the needle-making literature to define tip morphology using mathematical formulae:

1. Describing the tip apex curvature.
2. Describing the needle tip profile by the shape of the negative space around it. This is mostly used when describing concave tip shapes.

Parabolas and hyperbolas are common formulae used to describe needle tip morphology. nAPCI needle tips are shown with overlaid parabolas (left column) and hyperbolas (right column) for visual determination of best fit. In both definitions, nAPCI needles are parabolic. Desmos was used to generate plots.

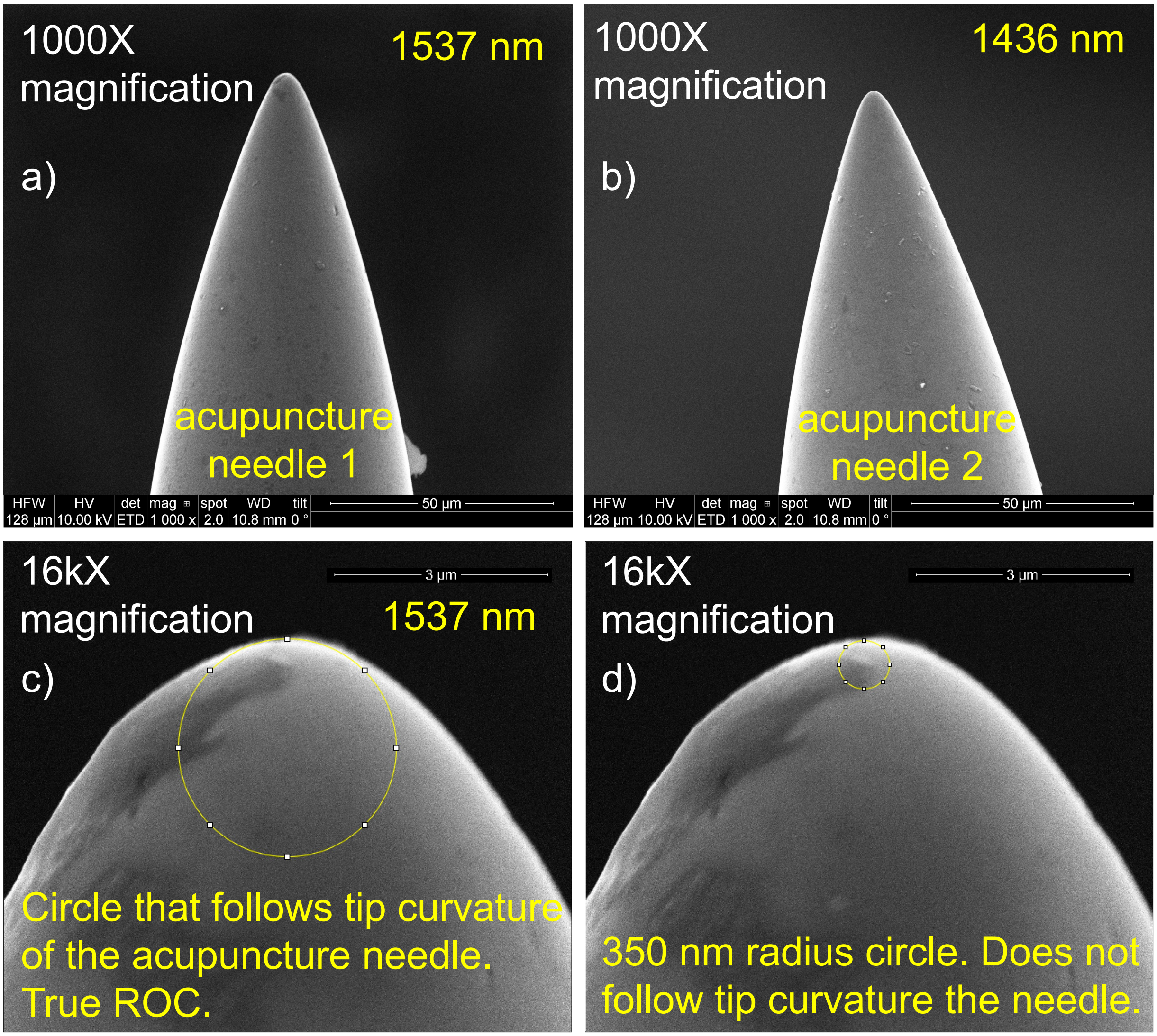

Figure S6. SEM images of SEIRIN Type J acupuncture needles which were reported in the literature to have an ROC of 350 nm. a) and b) depict two individual needles consistent in size and shape. The lower row depicts the leftmost needle tip at a higher magnification. When calculating ROC of a needle, a circle following the tip curvature is drawn in ImageJ. The radius of this circle is the ROC. c) depicts a circle that follows the tip curvature, giving a true ROC of 1.5 µm. d) shows the same image but with a 350 nm circle for visual comparison. It is apparent that SEIRIN acupuncture needles from this batch have ROCs significantly larger than the reported 350 nm. Calculated ROC is given in yellow and image magnification is given in white.

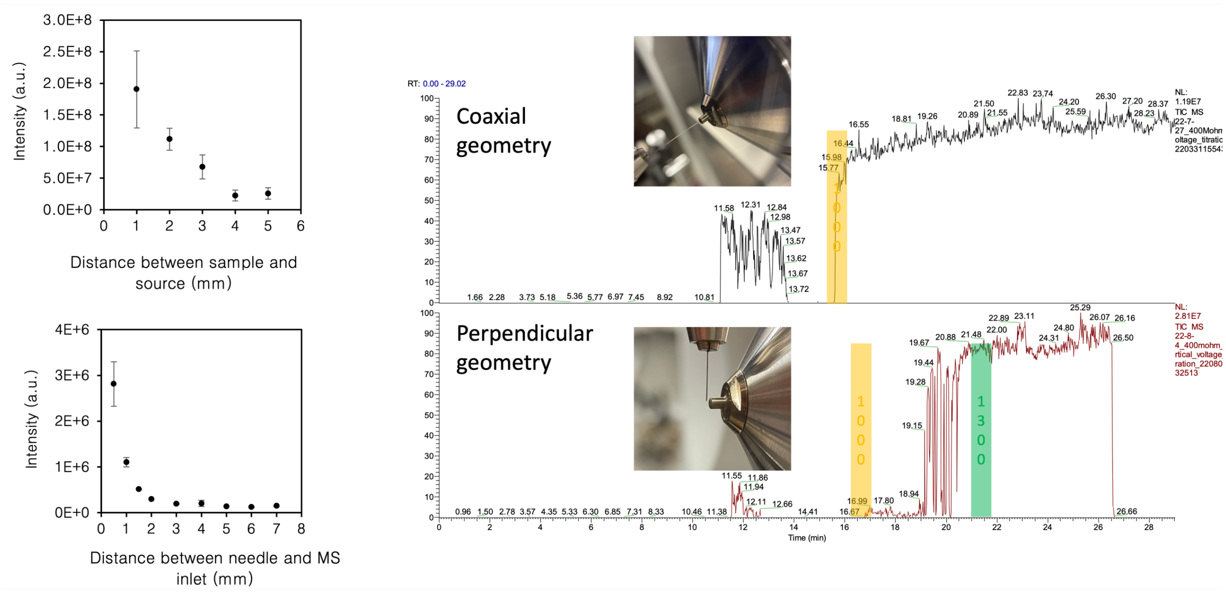

Figure S7. (left) nAPCI-MS geometry optimizations for the distance between the sample and source and the distance between the needle tip and the mass spectrometer inlet. (right) TICs produced by voltage titration testing to compare vertical and coaxial needle placement. Both needle tips were 0.5 mm away from the mass spectrometer inlet. Select voltages throughout titrations are labeled with colored rectangles (1000 V- yellow, 1300 V- green).

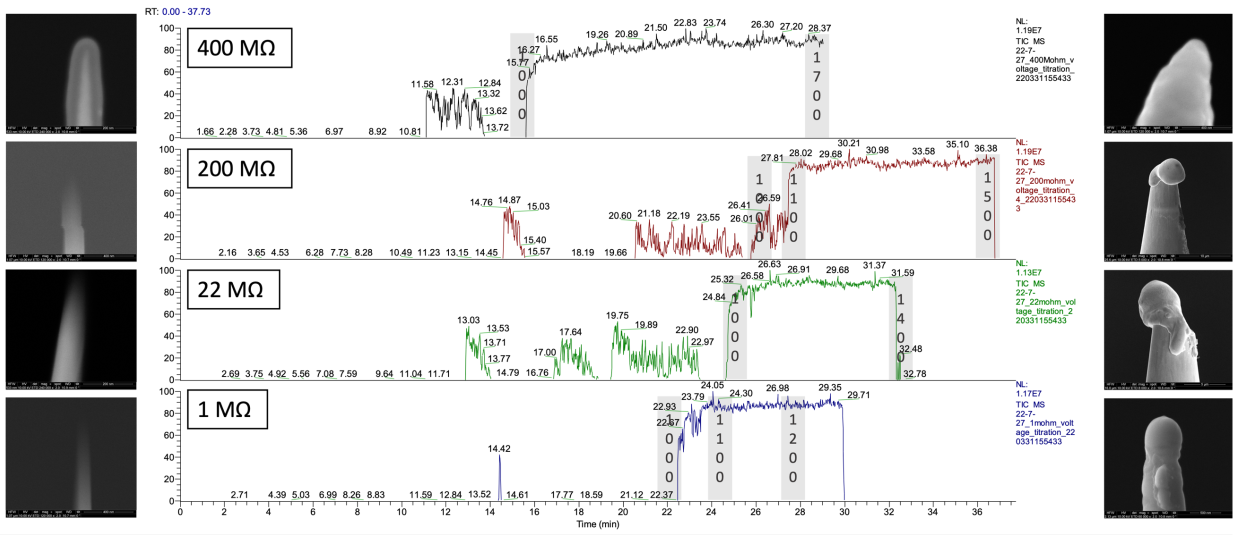

Figure S8. Voltage titrations for determining what resistance to use in the nAPCI circuit (in series between the power supply and nAPCI needle). Depicted via TICs. An SEM image taken before titration is located to the left of each TIC (120,000X – 240,000X magnification). An SEM image taken after titration is located to the right of each TIC. The lowest three resistances arced, and their needles appear to have melted (5,000X – 60,000X magnification). The needle used with the highest resistance (400 MΩ) remains relatively sharp after voltage titration (120,000X magnification). Select voltages throughout titrations are labeled with gray rectangles (in volts).

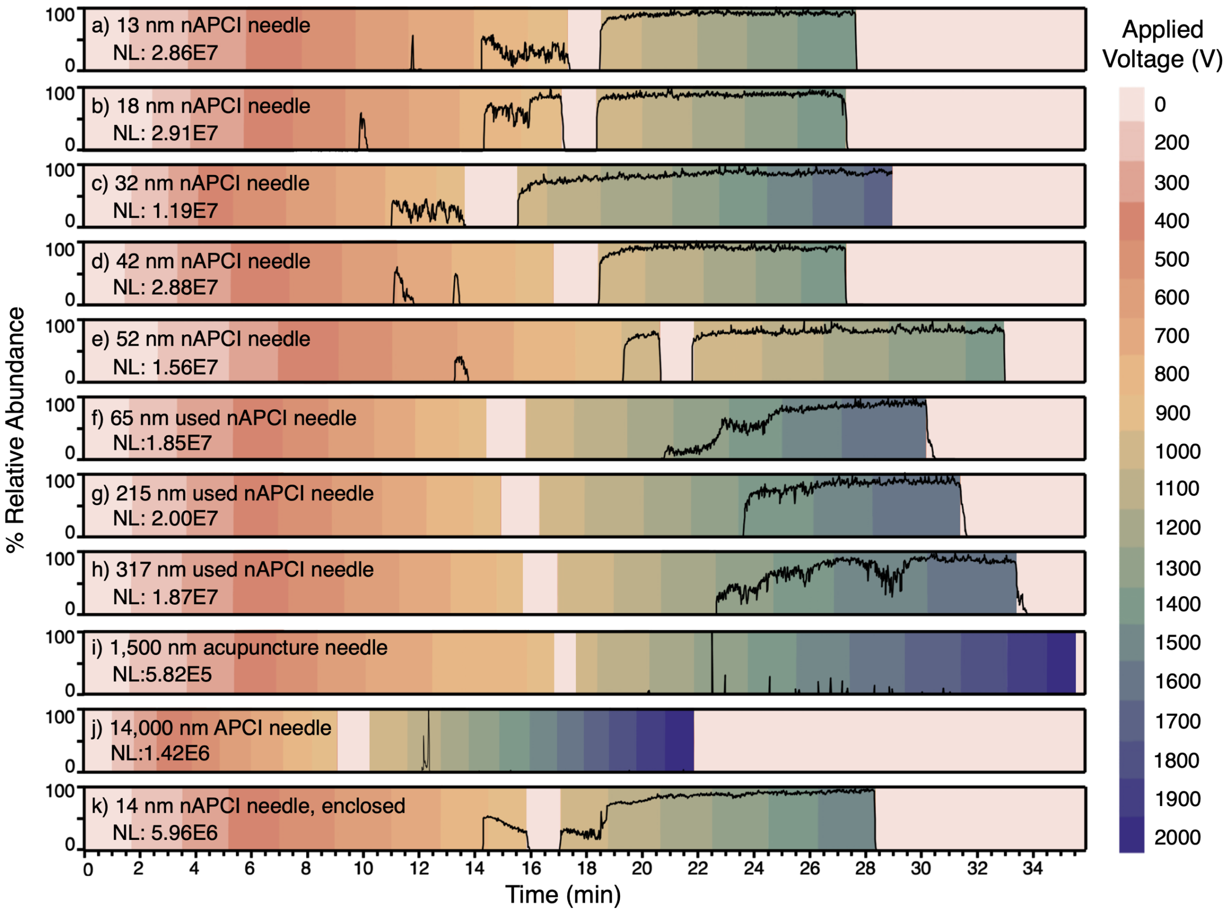

Figure S9. Depicted via total ion chromatogram (TIC): (a)-(e) Voltage-response over time of nAPCI needles in the exposed configuration, with ROCs of 13, 18, 32, 42, and 52 nm, respectively. This covers the full range of nAPCI needle sharpness. They all formed a stable corona at 1.0 kV. (f)-(h) Voltage-response over time of duller tungsten needles in the exposed configuration, with ROCs of 65, 215, and 317 nm, respectively. They required higher applied voltages to produce a stable corona. (i) Voltage-response over time of a commercial APCI needle in the exposed configuration with 14 µm ROC. (j) Voltage-response over time of an acupuncture needle in the exposed configuration with 1.5 µm ROC. They were both titrated up to 2000 V, and while they both produced fleeting flickers of low signal, neither was able to produce a stable corona discharge within this voltage range. (k) Voltage-response over time of an nAPCI needle in the enclosed configuration with 14 nm ROC. Overlaid color is indicative of applied voltage. At the start of each interval, the voltage was held constant for approximately 1 minute before ramping up to the next interval.

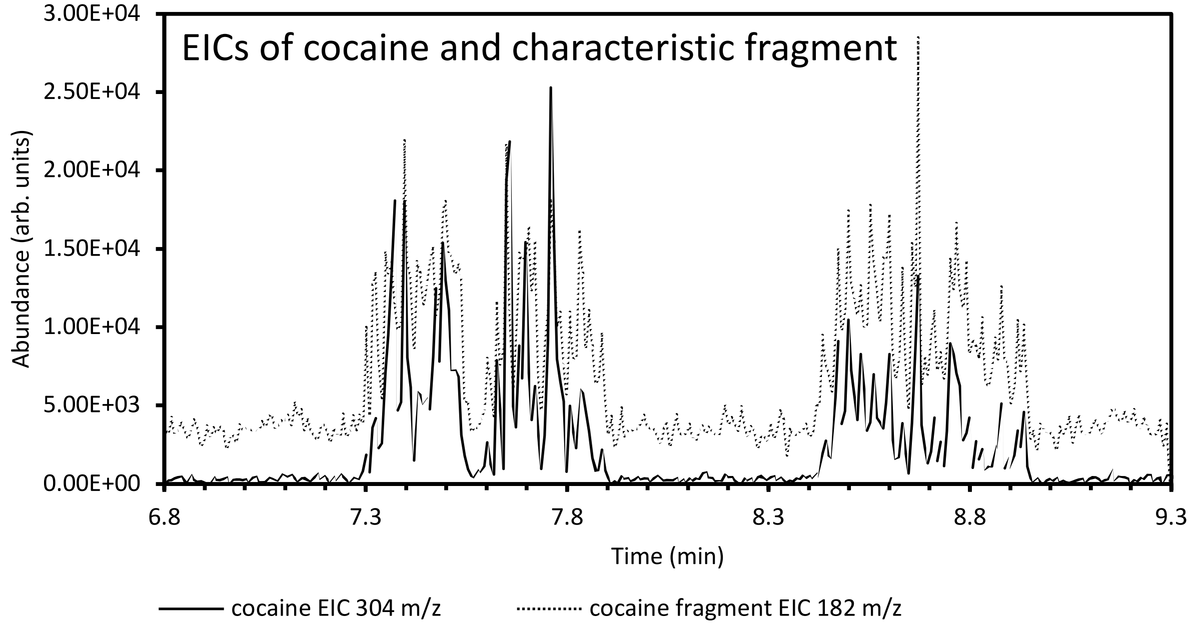

Figure S10. Overlaid extracted ion chromatograms (EICs) of cocaine (304 m/z) and characteristic fragment of cocaine (182 m/z) on the surface of American paper currency. They are present in the same time periods (when the bill is presented to the nAPCI source), confirming the presence and detection of cocaine.

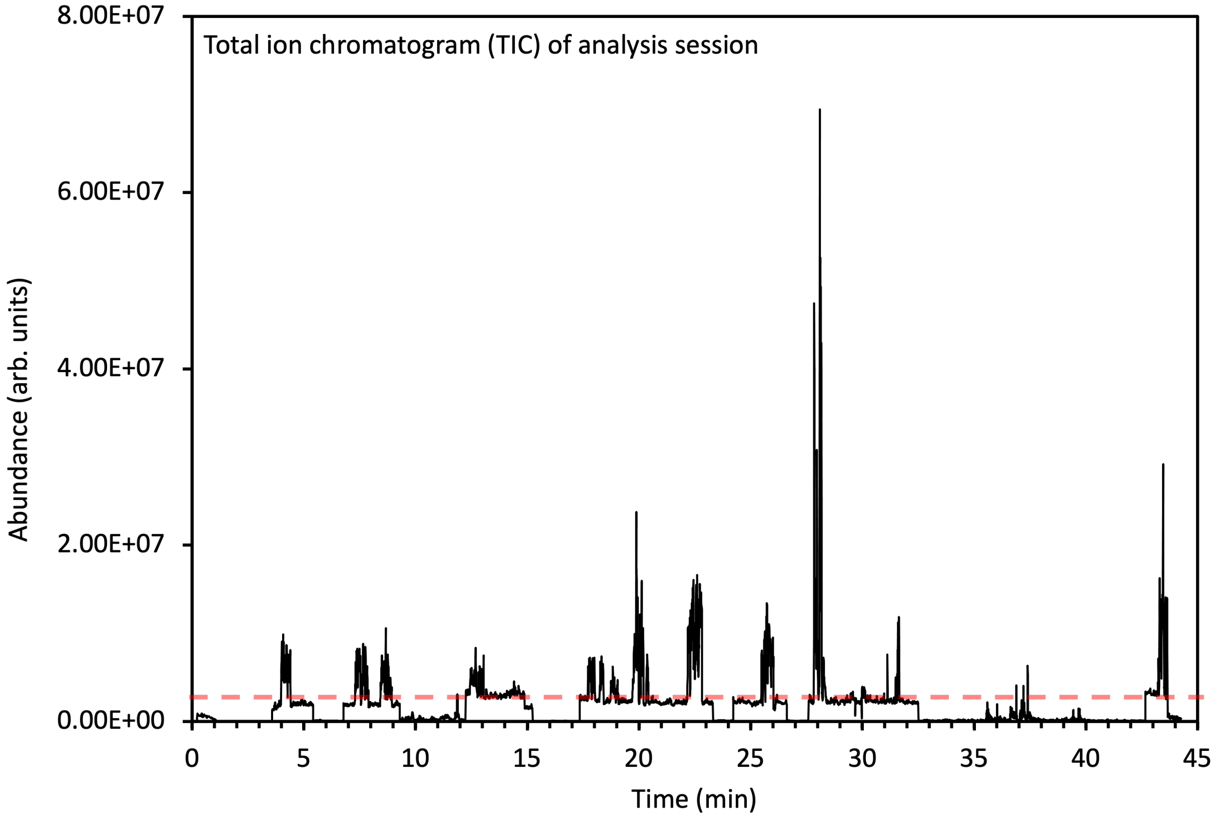

Figure S11. TIC of a 45-minute analysis session using nAPCI ionization in the exposed configuration. The baseline is highlighted by the red dashed line. Spikes in signal occurred when samples were analyzed. Regions of low signal in the middle of the session occurred when the instrument was changed to MS^2 or single ion monitoring mode. At the end of the session, the signal dropped off due to the power supply being turned off. The baseline remained consistent for the entire session.

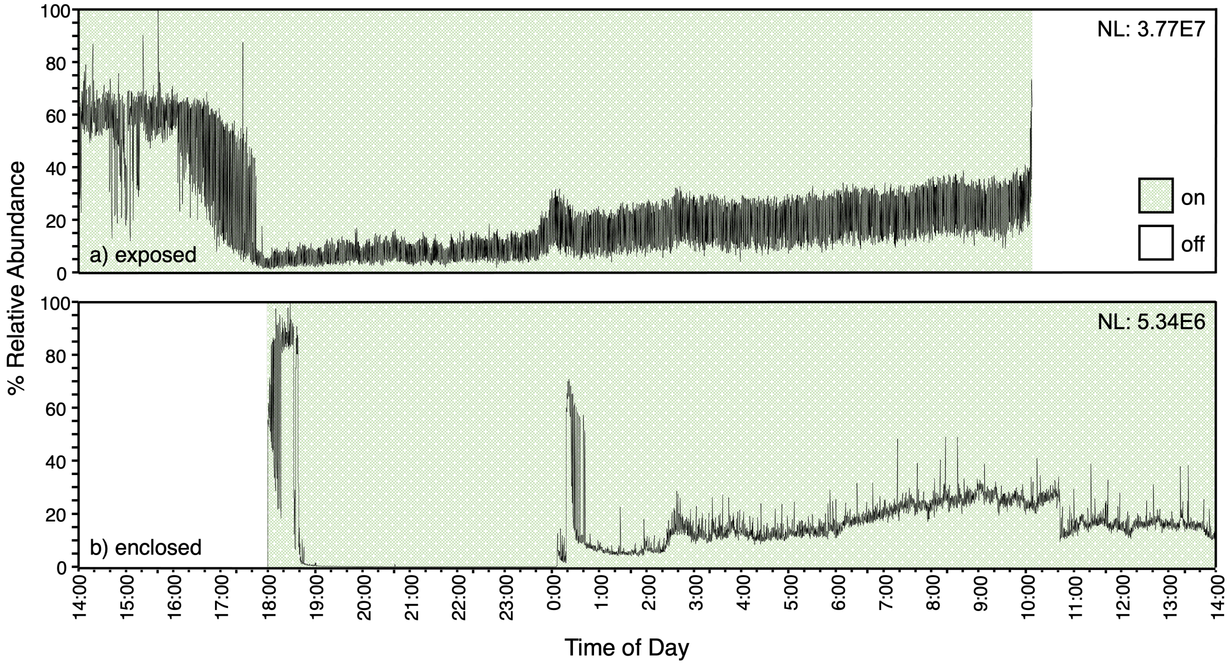

Figure S12. Indoor air monitoring TICs produced by nAPCI ionization in the (a) exposed and (b) enclosed configurations running for 20 hours continuously. The experiments were conducted on two different days. Each configuration was run at its respective operating voltage. Start times were staggered, as indicated by green sections. Analogous features include high initial signal until a drop off occurred at 6/7 PM, followed by hours of low signal until a spike occurred at midnight. From there, signal steadily rose until 10 AM, with a small spike at 2:30 AM.

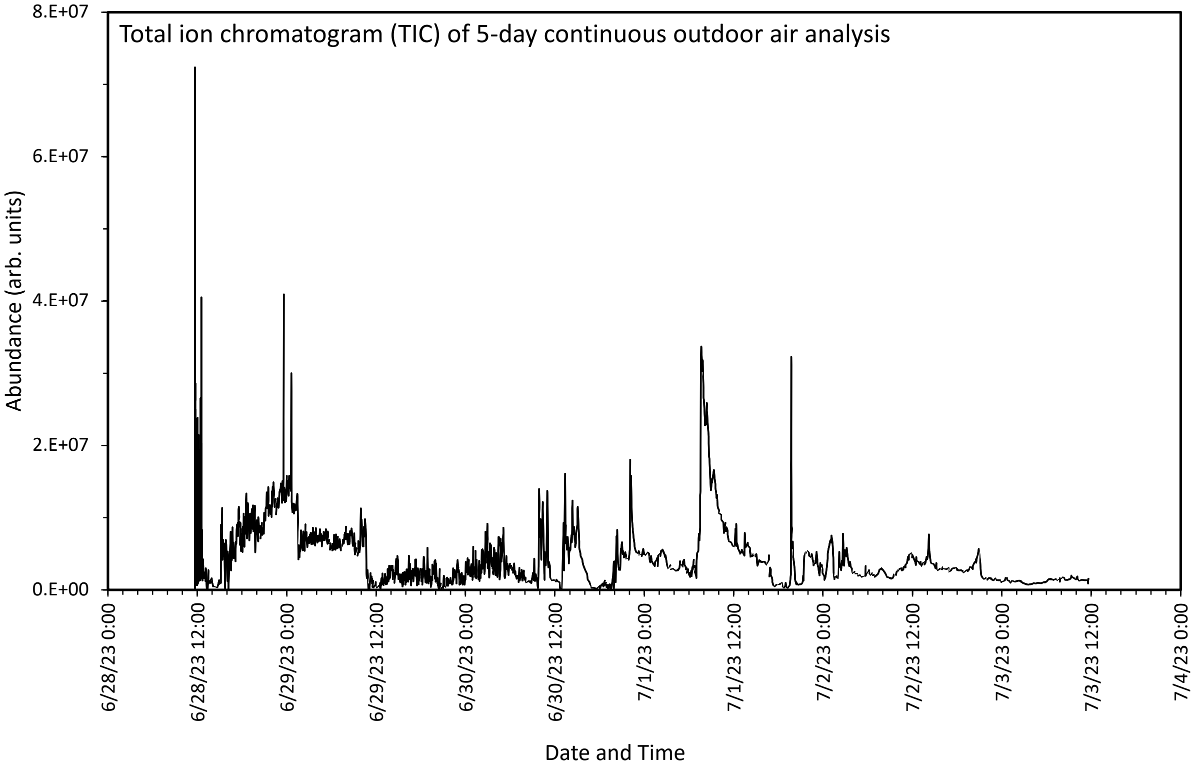

Figure S13. TIC of a 5-day outdoor air monitoring experiment using nAPCI ionization in the enclosed configuration with a 9 meter sniffing tube extending out of a nearby window. The experiment was started on 6/28/23 at 12:00, where signal begins. Signal remains present when the experiment is cut off at the 5 day mark.
